# Recent Advances in the Study of Diabetic Cardiac Autonomic Neuropathy

**DOI:** 10.31083/RCM45579

**Published:** 2026-01-12

**Authors:** Nan Wang, Jie Zhang

**Affiliations:** ^1^Department of Endocrinology, Ningbo No.2 Hospital, 315000 Ningbo, Zhejiang, China

**Keywords:** diabetes, cardiac autonomic neuropathy, early diagnosis, heart rate variability, treatment

## Abstract

Diabetic cardiac autonomic neuropathy (DCAN) is a common and serious complication of diabetes, and its early diagnosis and treatment are important for preventing cardiovascular events. At present, its diagnosis is mainly based on multiple functional investigations, such as heart rate variability (HRV) and cardiovascular reflex test. However, these methods are cumbersome to perform, time-consuming, and readily affected by patient cooperation and operator technique, resulting in limited clinical application. More importantly, DCAN still lacks standardized early diagnostic criteria and specific biomarkers. In recent years, the integration of multi-index diagnosis such as HRV, electrocardiograms (ECGs), continuous glucose monitoring (CGM) and machine-learning algorithms has improved the accuracy of early screening and prognosis. Here, we systematically review the latest research progress in relation to the pathological mechanism, diagnosis and treatment of DCAN, with a focus on novel biomarkers, therapeutic targets, and the potential for individualized treatment. This review provides new insights into DCAN, as well as the basis for early diagnosis and precise intervention.

## 1. Introduction

Diabetic cardiac autonomic neuropathy (DCAN) is a serious complication of diabetes and is independently associated with cardiovascular events, incidence and mortality. As the global prevalence of diabetes continues to rise, the research focus on DCAN has also increased significantly in recent years [1]. Epidemiological studies have shown that the prevalence of DCAN varies significantly between patients with type 1 diabetes (T1DM) and those with type 2 diabetes (T2DM), influenced mainly by various clinical features and demographic factors. In T1DM patients, chronic hyperglycemia is the principal risk factor driving the development of DCAN. In contrast, the pathogenesis of DCAN in T2DM patients is complicated by the cumulative effects of concomitant metabolic derangements—namely obesity, hypertension, and dyslipidemia. It is worth noting that insulin resistance, which is the underlying cause of T2DM and metabolic syndrome, plays a direct role in the onset of DCAN [1, 2].

DCAN often presents as asymptomatic, or with indistinct symptoms in the early stage, which greatly limits the effectiveness of treatment. Early diagnosis of DCAN is therefore crucial, especially when intervention is performed during the reversible stage of the disease [2]. The decrease in heart rate variability (HRV) is the earliest clinical indicator of subclinical DCAN. Diagnostic methods for DCAN include the cardiovascular autonomic reflex test, analysis of HRV, 24-h monitoring of blood pressure, baroreflex sensitivity test, and cardiac sympathetic nerve imaging. However, these methods are mostly limited to the research environment, with their wider application in clinical practice still facing many challenges [2, 3, 4]. In-depth studies on the epidemiological characteristics, pathogenesis, diagnostic methods, and treatment strategy of DCAN therefore have major clinical significance. Early diagnosis and intervention can improve the prognosis of patients, reduce the incidence of cardiovascular events and the mortality rate, and improve the patients’ quality of life [2, 5]. Intensive glycemic control is the main strategy used to prevent DCAN in patients with T1DM. To delay disease progression and improve the quality of life for patients with T2DM, more comprehensive management strategies are required, including improvement of microcirculation, neurotrophic support, drug therapy, and lifestyle intervention [2]. Although progress has recently been made in understanding the pathogenesis of DCAN, there is still no clear treatment that specifically targets this condition. Future studies should aim to develop novel therapeutic drugs that can alter the natural progression of the disease, and even reverse its course. In addition, further research is needed into the role of social determinants in the etiology of DCAN so that more targeted interventions can be developed [2, 5].

## 2. Pathophysiological Mechanisms

As one of the important complications of diabetes mellitus, the pathophysiological mechanisms underlying DCAN involve multiple complex molecular and cellular processes. Although the exact mechanisms have yet to be fully elucidated, the main mechanisms can be divided into the categories described below, based on results from existing studies.

### 2.1 Metabolism and Oxidative Stress

Persistent hyperglycemia activates the polyol pathway, hexosamine pathway, and protein kinase C (PKC), leading to excess production of advanced glycation end-products (AGEs). This metabolic abnormality causes dysfunction of the electron transport chain in mitochondria, generating reactive oxygen species (ROS) and triggering oxidative stress. Soriano *et al*. (2001) [6] showed that ROS not only causes direct damage to the DNA, proteins and lipids of neurons and Schwann cells, but also activates DNA repair enzymes such as poly ADP-ribose polymerase (PARP), leading to cellular energy depletion and apoptosis. The accumulation of ROS also promotes atherosclerosis through the formation of oxidized low-density lipoprotein (ox-LDL), further exacerbating injury to blood vessels and nerves [7, 8].

### 2.2 Hypoxia and Sleep Apnea

Studies by Riley *et al*. [9] and Javaheri *et al*. [10] have shown that obstructive sleep apnea (OSA) is common in diabetes patients. OSA induces activation of the hypoxia-inducible factor-1α (HIF-1α) pathway through intermittent hypoxia, causing oxidative stress and excessive activation of the sympathetic nerve, which then has a synergistic effect on the occurrence and development of DCAN [9, 10, 11]. Baessler *et al*. [12] also confirmed through meta-analysis that OSA-related chronic intermittent hypoxia can directly promote systemic inflammatory reactions and exacerbate autonomic nerve function loss of balance [13]. These results indicate that intervention measures targeting OSA could potentially have important clinical value for the prevention and treatment of DCAN [12, 13].

### 2.3 Inflammatory Factor Pathway

The inflammatory factor pathway plays a key role in promoting the development of DCAN. A study by Egaña-Gorroño *et al*. [7] revealed this pathway can induce the overexpression and release of various pro-inflammatory cytokines, especially Tumor Necrosis Factor-alpha (TNF-α), Interleukin-1β (IL-1β) and Interleukin-6 (IL-6), forming a chronic low-grade inflammatory microenvironment. This directly induces neuron apoptosis and Schwann cell dysfunction, as well as damaging the neurovascular unit and exacerbating oxidative stress, ultimately leading to impaired function of cardiac autonomic innervation [3, 6, 7, 14]. Inflammatory factors can also form a positive feedback loop with the renin-angiotensin-aldosterone system (RAAS) and the sympathetic nervous system, amplifying each other and further promoting myocardial fibrosis, cardiac ventricle remodeling, and autonomic nervous loss of balance, thereby accelerating the progression of DCAN [2, 3, 14].

### 2.4 Collagen Metabolism and Fibrosis Pathway

The research team of Frimodt-Møller and Hansen [15] reported that collagen markers are closely associated with neuropathy in patients with T1DM [5]. By investigating the formation marker of collagen type VI (COL6), namely pro-collagen VI (α3 chain) C-terminal propeptide (PRO-C6), and the degradation marker of collagen type III (COL3), namely collagen type III metabolite (C3M), they determined that metabolic abnormality of collagen increased the accumulation of extracellular matrix around nerves, as well as having potential pro-inflammatory and pro-fibrotic effects, thereby impairing nerve function [5, 16, 17]. This finding provides a potential new target for the future treatment of diabetic neuropathy.

### 2.5 Mitochondrial Dysfunction

Sajic *et al*. [18] reported that a high fat diet reduces the mitochondrial membrane potential of axonal mitochondria and impairs the conduction ability of sensory neurons at physiological frequencies. This diet also reduces the Ca^2+^ level in sensory axons, increases mitochondrial elongation, and upregulates expression of the key regulatory factor Peroxisome Proliferator-Activated Receptor Gamma Coactivator 1-Alpha (PGC1α) [19, 20], further exacerbating the loss of balance in energy metabolism and leading to impaired nerve impulse conduction. These results suggest that mitochondrial dysfunction may be an important mechanism leading to abnormal neurological function early in diabetes [18, 20].

### 2.6 Purinergic Signaling Pathway Abnormalities

The purinergic signaling pathway plays an important role in various cell types, with recent studies also demonstrating that purinergic receptors are involved in the pathogenesis of DCAN [21]. In the diabetic state, sympathetic nerve injury in the heart can affect expression of purinergic receptors. The activated purinergic receptors can in turn influence the phosphorylation of different signaling pathways and the regulation of inflammatory processes. For example, increased expression of P2X3 receptor (P2X Purinoceptor 3) in the cervical sympathetic nerve ganglia promotes the release of inflammatory factors such as IL-1β and TNF-α, as well as activating the extracellular signal-regulated kinase 1/2 (ERK1/2) signaling pathway, thereby exacerbating nerve injury [22, 23]. Increased expression of the P2X4 receptor (P2X Purinoceptor 4) in the stellate ganglia may be involved in the pathogenesis of neuropathic pain and peripheral inflammatory pain by activating microglia [24]. Additionally, increased expression of the P2Y12 receptor (P2Y Purinoceptor 12) in satellite glial cells of the sympathetic nerve ganglia can further promote the release of pro-inflammatory cytokines and exacerbate the inflammatory reaction [25, 26].

## 3. Diagnostic Methods

DCAN mainly affects functions of the heart, blood vessels, gastrointestinal tract, genitourinary system, and pupil that are regulated by the autonomic nervous system. Its clinical manifestations are diverse, with cardiovascular symptoms being particularly prominent, including resting tachycardia, exercise intolerance, as well as symptoms related to orthostatic hypotension, such as dizziness, blurred vision, neck pain, and syncope [1, 27].

The Toronto Neuropathy Experts have emphasized the importance of the Cardiovascular Autonomic Reflex Test (CART) and regard it as the “gold standard” for diagnosing DCAN [5]. CART evaluates the functions of parasympathetic and sympathetic nerves through a series of tests, including deep breathing, the Valsalva maneuver, HRV during supine and standing positions, and the supine-to-standard blood pressure test (see Table 1). These tests provide important information about cardiac autonomic nerve function, thus helping doctors to diagnose DCAN more accurately [27].

**Table 1.** **Cardiovascular autonomic reflex test (CART)**.

Category	Item	Specific methods and standards	Description/significance
Screening objects	High-risk population	Diabetes patients with microvascular and neurological complications	These patients should undergo assessment of symptoms and signs of DCAN.
		Patients with asymptomatic hypoglycemia	
Screening methods	Gold standard (CART)	Cardiovascular Autonomic Reflex Tests, including:	Reflect parasympathetic and sympathetic nerve functions.
		1. Deep breathing HRV	
		2. Valsalva maneuver HRV	
		3. Supine-standing HRV	
		4. Supine-to-standard blood pressure test	
	Other methods	1. HRV analysis	Aid in diagnosis.
		2. Hemodynamometry during positioning changes	
		3. 24 h blood pressure ambulatory monitoring	
Diagnostic basis	Clinical symptoms	Palpitation, dizziness, asthenia and weakness, visual impairment, syncope, etc.	Diagnosis should be combined with clinical symptoms and/or physical examination.
	Abnormal signs	1. Resting Tachycardia	
		2. Orthostatic Hypotension	
		3. Decreased HRV	
Detailed test criteria	1. Supine-to-standard blood pressure test	Systolic blood pressure decreased ≥20 mmHg within 3 minutes after changing from supine to upright position. Considered as orthostatic hypotension	
	2. 24-h blood pressure ambulatory monitoring	Applicable to patients suspected of having loss of circadian change in blood pressure	
	3. Resting Tachycardia	Heart rate >100 beats/min at rest	
	4. HRV detection	Deep breathing HRV: Perform deep breathing at a frequency of 6 times/min, and calculate the difference between the fastest heart rate during inspiration and the slowest heart rate during expiration (I-E)	Normal: Heart rate varies greatly, with high HRV.
		Orthostatic HRV: Calculate the ratio of the longest to the shortest RR interval after standing (30:15 ratio)	CAN patients: Heart rate shows no change, with decreased HRV.
		Valsalva maneuver HRV: Perform Valsalva maneuver, and calculate the maximum RR interval/minimum RR interval (Valsalva ratio)	Note: Avoid performing Valsalva maneuver on patients with proliferative retinopathy.
Diagnostic classification	Possible/early-stage DCAN	One abnormal HRV result or two or more borderline results	
	Confirmed DCAN	At least two abnormal HRV results	
	Severe/advanced stage DCAN	Abnormal HRV results and presence of orthostatic hypotension	

Note: 1 mmHg = 0.133 kPa. HRV, heart rate variability.

Although CART is widely regarded as the “gold standard” for diagnosing DCAN, this method also has some limitations and drawbacks. It includes multiple steps and tests, such as deep breathing, the Valsalva maneuver, supine-to-standing HRV, and supine-to-standard blood pressure test, all of which require operation and interpretation by professionals. This complex and time-consuming process is unlikely to be suitable for rapid screening or for the assessment of large populations. Moreover, the implementation of CART requires professional equipment such as cardiac autonomic function testing systems, which may limit its application in medical institutions with limited resources or insufficient equipment. Therefore, in recent years the clinical community has been searching for simpler, faster, and more accurate methods to optimize the diagnostic process.

HRV analysis is an important non-invasive tool discovered in recent years for evaluating DCAN, with its value confirmed by multi-dimensional studies. Genetic studies suggest that HRV is regulated by specific genetic loci. Nolte *et al*. [28] identified multiple gene loci associated with HRV through genome-wide association analysis. These loci are also associated with the risk of cardiac disorder, indicating that HRV is not only an indicator of autonomic nerve function, but may also have a genetic basis and value for cardiovascular prognosis [28]. Zhang *et al*. [29] showed that combining the traditional symptom scoring tool Composite Autonomic Symptom Score 31 (COMPASS 31) with HRV parameters could significantly improve the diagnostic accuracy for DCAN in T2DM patients, reflecting the superiority of integrating multiple indicators. Recent advances in analytical methods have led to the successful application of machine learning (ML) technology for in-depth mining of HRV data. Alkhodari *et al*. [30] extracted HRV traits from 24-h dynamic ECG data and constructed a diagnostic model by combining with an ML algorithm. This could efficiently screen DCAN in diabetes patients with microvascular complications, thus providing an automated solution for large-scale population screening [30]. However, attention should be paid to the reliability of HRV measurements. Besson *et al*. [31] found that although strict control of the test environment and positioning was associated with high clinical reliability of short-term HRV measurement, the results could be affected by test conditions. This suggests that standardized detection procedures are crucial for ensuring the accuracy of screening results.

The scope of applications for HRV is continuously expanding. In addition to its use in evaluating autonomic nerve function, HRV is also associated with mental and psychological disorders such as anxiety. Tomasi *et al*. [32] explored its potential as a biomarker for anxiety disorder. These authors suggested that attention should be paid to the potential impact of psychological factors on HRV results in diabetes patients [32]. Besides HRV, other ECG indicators such as the QT interval have also been used in DCAN assessment. Vasheghani *et al*. [33] found the QT interval index is significantly correlated with DCAN and can serve as an effective supplementary diagnostic indicator in addition to HRV.

Beyond HRV, additional physiological indices can also play a pivotal role in the diagnosis of DCAN. Alkhodari *et al*. [30] demonstrated the potential of ML by using demographic, clinical, and laboratory data to screen for T2DM microvascular complications, further demonstrating the AI-led development of diabetes management. Sudoscan is an innovative medical device that assesses sweat gland function in a non-invasive manner, thereby providing a new technical approach for the evaluation of diabetic autonomic neuropathy [34].

In the field of imaging-assisted diagnosis, the Silesia Diabetes Heart Study by Nabrdalik *et al*. [35] demonstrated that AI-based classification of DCAN from retinal fundus photographs offers a novel imaging biomarker and diagnostic paradigm for this condition. Moreover, Jaiswal *et al*. [36] found that impaired cardiovascular autonomic nerve function in T1DM patients was significantly associated with the occurrence of severe hypoglycemia events. This suggests that autonomic nerve regulation disorders may weaken the body’s normal compensatory response to hypoglycemia, thereby increasing the risk [36]. Glycemic variability (GV) itself is also considered an important driving factor for the progression of neuropathy. Zhang *et al*. [37] systematically reviewed GV and noted that it can promote nerve injury by mechanisms such as exacerbation of oxidative stress and inflammatory reactions. Therefore, the analysis of GV parameters derived from continuous glucose monitoring (CGM) is not only helpful for the refinement of blood glucose management, but may also be an additional reference for the diagnosis and risk stratification of DCAN [37]. In conclusion, the current diagnosis of DCAN integrates multi-dimensional information that spans traditional functional assessment to novel AI image recognition, and autonomic nerve-specific detection to blood glucose system evaluation. This is a reflection of the general trend toward multimodality and AI.

## 4. Treatment

### 4.1 Lifestyle Intervention

Lifestyle intervention occupies a central position in the treatment and management of DCAN. Reasonable nutritional intake and appropriate exercise are the basis for improving the condition of DCAN patients. Although no specific dietary pattern is recommended, the Mediterranean diet, low carbohydrate diet, and low fat diet all have potential benefits for diabetes patients [37].

Table 2 (Ref. [1, 37, 38, 39, 40]) presents the stratified management of DCAN based on body mass index (BMI) and the severity of disease. This approach accurately formulates exercise and dietary recommendations, maximizes therapeutic benefits while ensuring safety, improves metabolic disorders, reduces oxidative stress, and protects autonomic nerve function [37, 38, 39].

**Table 2.** **Recommendations for lifestyle interventions in patients with diabetic cardiac autonomic neuropathy (DCAN) based on BMI classification and disease severity**.

Stratification index	Stratification trait	Kinesitherapy	Dietary therapy	Points for attention
Based on BMI classification				
Overweight/obese (BMI ≥24 kg/m^2^)	Excessive weight, often accompanied by grade III insulin resistance	Goal: 5%–10% weight loss	Goal: low-calorie balanced diet	Avoid high-intensity exercise to prevent joint injury; exercise should be combined with diet to ensure weight loss effect
		• Aerobic exercise (brisk walking, swimming) for 150–300 minutes per week	• Strictly control total calories to create a reasonable calorie deficit	
		• Combined with resistance training twice a week	• Prioritize foods with low glycemic index (GI) and high fiber, and ensure high-quality protein [37, 39]	
		• Intensity: start with low to moderate intensity (50–60% maximum heart rate) [39]		
Normal weight (BMI 18.5–23.9 kg/m^2^)	Normal weight, but there may be abnormal body composition	Goal: Improve body composition and maintain weight	Goal: Balanced nutrition and stable blood glucose	Pay attention to muscle content; resistance training is crucial for preventing sarcopenia [40]
		• Equal emphasis on aerobic exercise (150 minutes per week) and resistance training (2–3 times per week)	• The Mediterranean diet pattern is recommended, which is rich in antioxidants and Omega-3 fatty acids	
		• Intensity: Moderate intensity (60–70% of maximum heart rate) [38, 39]	• Focus on dietary quality and avoid hidden sugars [1, 38]	
Based on the severity of DCAN				
DCAN early/subclinical stage	Abnormal HRV, asymptomatic	Goal: Increase vagal tone and delay progression	Goal: Reduce glucose variability (GV)	Cardiac stress test is not required before exercise, but cardiac autonomic nerve function should be reviewed regularly [1]
		• Regular aerobic exercise, ≥150 minutes per week, moderate intensity	• Strictly adopt a low GI diet to reduce postprandial blood glucose fluctuations	
		• Combine with mind-body exercises such as tai chi and yoga [1, 37]	• Increase intake of foods rich in neurotrophic nutrients [37, 39]	
DCAN advanced stage/clinical stage	Presence of resting tachycardia, postural hypotension, etc.	Goal: Avoid triggering events and maintain function	Goal: Manage symptoms and prevent malnutrition	Cardiovascular evaluation must be performed before exercise, and attention should be paid to the risk of syncope caused by exercise or postural changes [1, 39]
		• Exercise extreme caution; rehabilitation under monitoring is preferred	• If there is no Contraindication, Sodium salt and fluid intake can be appropriately increased to cope with hypotension.	
		• Low-intensity daily activities are recommended; high-intensity exercise is strictly prohibited [1, 39]	• Eat small, frequent meals to avoid postprandial hypotension [1, 39]	

BMI, Body Mass Index.

### 4.2 Blood Glucose Control

Serhiyenko VA and Serhiyenko AA [1] reported that hyperglycemia was a key risk factor for DCAN by directly causing injury to autonomic nerve fibers that innervate the heart. In addition, it damages the myelin sheath structure through multiple mechanisms such as the induction of oxidative stress, accumulation of AGEs, and activation of the polyol pathway. Active and strict blood glucose control is therefore regarded as a key factor in preventing and delaying the course of DCAN [1, 39]. The landmark long-term follow-up study, Diabetes Control and Complications Trial/Diabetes Intervention and Complications Epidemic (DCCT/EDIC), provides the highest level of evidence-based support for this [40]. The DCCT/EDIC study confirmed that early implementation of intensive glycemic control in patients with diabetes cannot completely reverse neuropathy in the short-term. However, it can significantly reduce the long-term risk of neuropathy and bring about a sustained “metabolic memory” benefit. In other words, appropriate early blood glucose management can continue to exert cardiovascular protective effects in subsequent decades.

In terms of specific blood glucose management strategies, a study by Evans and Li [38] revealed the underlying molecular mechanisms involved and suggested potential therapeutic directions. Their research indicated that persistent hyperglycemia can trigger pathological remodeling of intrinsic cardiac nerve ganglia, including neuron apoptosis and neuroglia dysfunction. Intensive glycemic control (usually referred to as glycosylated Hemoglobin [HbA1c] <7%) can effectively inhibit these harmful pathways and reduce neuroinflammation and oxidative injury, thereby protecting neural structures. However, for elderly patients with a long medical history or with severe complications, overly aggressive hypoglycemic therapy can increase the risk of hypoglycemia. Recurrent episodes of hypoglycemia can also worsen autonomic nervous dysfunction by activating the sympathetic nervous system [1]. Therefore, modern treatment concepts emphasize the safe achievement of optimal blood glucose control through individualized treatment. A comprehensive management plan is usually adopted, including CGM, insulin pump (continuous subcutaneous insulin infusion [CSII]), and new hypoglycemic drugs such as GLP-1 receptor agonists and SGLT2 inhibitors. In addition to lowering blood glucose, these drugs may also confer independent benefits through cardiovascular and nerve protection, providing new multi-target possibilities for the prevention and treatment of DCAN [1, 38].

### 4.3 Drug Therapy

#### 4.3.1 Lipid-Lowering Drugs

The treatment of diabetic dyslipidemia (DLP) is an important part of DCAN management. Statins block the cholesterol synthesis pathway in the liver by inhibiting 3-Hydroxy-3-Methylglutaryl-Coenzyme A (HMG-CoA) reductase, thereby reducing cholesterol levels. For example, the Medical Research Council/British Heart Foundation (MRC/BHF) Heart Protection Study (HPS) found that statins significantly reduced cardiovascular risk in diabetic patients. Moreover, when combined with the maximum dose of statins, Proprotein Convertase Subtilisin/Kexin Type 9 (PCSK9) inhibitors such as alirocumab and evolocumab further reduced low-density lipoprotein cholesterol (LDL-C) by approximately 60% [41].

#### 4.3.2 Correction of Vascular Endothelial Function

The correction of vascular endothelial dysfunction is another key aspect in the treatment of DCAN. Studies have shown that strict glucose control, lifestyle adjustments, and mitigation of risk factors can partially improve the indicators of DCAN [42]. For example, novel hypoglycemic drugs such as Sodium-Glucose Cotransporter-2 (SGLT-2) inhibitor and Glucagon-Like Peptide-1 Receptor Agonist (GLP-1RA) not only improve glucose control, but may also have a positive impact on DCAN by acting directly or indirectly on the autonomic nervous system. By reducing norepinephrine expression in the kidney and improving renal hemodynamics, SGLT-2 inhibitor has a positive impact on cardiovascular outcomes independent of its effect on glycosuria. Moreover, while GLP-1RA increases the heart rate and may have an impact on HRV, its positive effects on cardiovascular mortality are also worth noting [43]. The cardiovascular protective effects of these novel therapies may be related to their potential impact on the autonomic nervous system, especially in DCAN patients where these drugs are an important component in reducing cardiovascular risk. Therefore, in addition to traditional blood glucose control measures for diabetes mellitus, the protection and correction of vascular endothelial function and the autonomic nervous system should also be considered in the comprehensive management of DCAN. This should lead to improved clinical prognosis for patients with DCAN.

### 4.4 Biological Factors

Studies have revealed that the development of DCAN is not caused by a single pathway, but is instead driven by a microenvironment composed of pro-inflammatory cytokines (e.g., TNF-α, IL-1β, IL-6), deficiency of neurotrophic factors (e.g., nerve growth factor [NGF], brain-derived neurotrophic factor [BDNF]), and excessive oxidative stress mediators (e.g., ROS) [38, 41, 44]. Therefore, targeting these key biological factors and correcting their loss of balance could be a highly promising treatment strategy for DCAN.

Research on biological factor-targeted therapy has mainly focused on two strategies: “inhibiting harmful factors” and “supplementing beneficial factors”. There is substantial evidence supporting antagonistic strategies that target harmful factors. For example, Fabiyi-Edebor [45] showed that exogenous supplementation with the antioxidant vitamin C can effectively neutralize excessive ROS and significantly improve HRV parameters in diabetic rat models, thus confirming the feasibility of protecting autonomic nerve function by reducing oxidative injury. In addition, Ziegler *et al*. [41] and Eleftheriadou *et al*. [42] found that biological agents such as monoclonal antibodies or small molecule inhibitors targeting core inflammatory pathways (e.g., TNF-α, IL-1β) showed good effects in other diabetic complication models, providing a theoretical basis for their use in treating DCAN. On the other hand, enhancing the neurotrophic support of beneficial biological factors is also another important direction. A study by Evans and Li [38] focused on upregulating the expression levels of endogenous NGF and BDNF through pharmacological or gene therapy approaches to promote neuron survival, axon regeneration and synaptic plasticity, thereby repairing damaged autonomic nerve pathways. Also of note, a study by Zaki *et al*. [43] confirmed that exercise intervention, as a pleiotropic non-pharmacological approach, can simultaneously regulate the two strategies described above. Exercise can improve the metabolic profile and autonomic nerve function of DCAN patients by reducing the level of inflammatory factors, enhancing the antioxidant defense capacity, and possibly also promoting the secretion of neurotrophic factors [43].

## 5. Conclusions

DCAN is a complex, multi-system disease. Although significant progress has recently been made in understanding the underlying pathological mechanism and improving diagnosis and treatment, several remaining key challenges and knowledge gaps urgently need to be addressed by future research. First, current diagnosis is still highly dependent on functional investigations such as HRV and the cardiovascular reflex test. The operational complexity of these tests and their dependence on standardized procedures have greatly limited their clinical application. Therefore, future research should focus on developing and validating novel biomarkers that are non-invasive, highly sensitive, and easily standardized. For example, body fluid detection systems based on collagen metabolism markers (e.g., PRO-C6, C3M) [5, 15], inflammatory factor profiles (e.g., TNF-α, IL-1β) or purinergic receptors (e.g., P2X3, P2Y12) [21, 22, 23, 24, 25, 26], combined with AI algorithms for the integrated analysis of multi-modal data (e.g., ECG ambulatory, CGM, retina images) [38] should enable the construction of more objective and reproducible models for early diagnosis. Moreover, these models should assist with DCAN typing, and stratified diagnosis and treatment systems.

At the treatment level, although new hypoglycemic drugs such as SGLT-2 inhibitor and GLP-1RA have shown cardiovascular protective potential, their specific intervention effects in DCAN and the mechanisms involved have yet to be elucidated. Randomized controlled trials with DCAN will be required in the future to determine whether these drugs can delay or even reverse the progression of DCAN by improving blood glucose fluctuation, inhibiting inflammatory reactions, or regulating autonomic nerve remodeling. In addition, precise treatment strategies that target specific mechanisms should also be investigated, such as targeted inhibition of harmful purinergic signaling pathways [26], the use of neurotrophic factors or antioxidants such as vitamin C [45] to improve neuron survival and function, and regulation of autonomic nerve balance through exercise intervention [43]. In summary, interdisciplinary integration and the convergence of innovative technologies should enable research encompassing “mechanism exploration to biomarker identification to individualized treatment”. This will be a key goal for achieving breakthroughs in the DCAN field, and ultimately for improving the long-term prognosis of diabetes patients.
